# Supplementary material for: Transcriptional immunogenomic analysis reveals distinct immunological clusters in paediatric nervous system tumours
Source: Genome Med. 2023 Sep 7;15:67. doi: 10.1186/s13073-023-01219-x (PMC10486055; doi:10.1186/s13073-023-01219-x)
Supplement: Supplementary file 1 — Additional file 1. Supplementary figures for the manuscript. [file 13073_2023_1219_MOESM1_ESM.pdf]

Supplementary figure legends

Figure S1. Characterization of the CBTN dataset. A) Sankey plot showing the CBTN samples and their annotations included in this study. **B-C)** Heatmaps showing hierarchical clustering of CBTN samples using top 1000 variable genes before (A) and after (B) removing samples that pathological annotations did not match their transcriptional clusters. **D-E)** Heatmaps showing hierarchical clustering using top 1000 variable genes for the CBTN ATRT (D) and medulloblastoma (E) samples to identify tumour subgroups.

Figure S2. Overall strategy for the present study and assessment of ESTIMATE performance using *in silico* simulations. A) Overview of data sources and types used in the present study and analysis strategy. **B)** Scatter plot and non-linear regression line showing correlation between the ESTIMATE Immune score and immune read percentage in simulated PDX samples with increasing percentage of immune reads.

Figure S3. Immunohistochemistry confirms variable immune infiltration in pedNST. A) Boxplots showing H-scores for anti-CD8, CD4 and CD19 staining across an independent cohort of pedNST. **B)** Representative images for immunohistochemistry. In all boxplots, boxes show median and IQR and whiskers represent 1.5 times IQR.

Figure S4. Analysis of immune deconvolution tools reveals discordance in immune inference. A-F) Heatmaps representing immune cell-type estimates derived from CIBERSORT (A), EPIC (B), TIMER (C), MCPCOUNTER (D), XCELL (E) and QUANTISEQ (F) across PDX and pedNST. Dendrograms show hierarchical clustering between cell-types (rows) and samples (columns). **G)** Barplots obtained from TIMER2 comparing estimates of 8 immune cell-types (columns) derived from six immune deconvolution tools (rows) in 10 PDX medulloblastoma samples (bars).

Figure S5. Adult tumour microenvironment clusters and tumour purity analysis confirm characteristics of immune clusters. A) Boxplot depicting tumour purity scores for 156 ICGC samples inferred from copy number estimates (ACEseq). Boxes show median and IQR and whiskers represent 1.5 times IQR. **B)** Representative plots showing B allele frequencies (BAF) and copy numbers (TCN) in each immune cluster.

Figure S6. Segmentation analysis of H&E images and methylation-based deconvolution analysis validate transcriptional immune clusters. A-B) Boxplots showing average TIL score based on segmentation analysis of pathological images across cancer entities (A) and tumour sites (B) using H&E images from CBTN. **C)** Boxplots showing average TIL score based on segmentation analysis of pathological images in samples with > 5% immune read percentage (left panel), in samples collected from cerebral hemisphere (right panel). **D)** Ridge plots illustrating z-score distributions for immune cell-types as estimated by methylation-based deconvolution analysis across immune clusters. Pairwise two-sided Student's t-test with Bonferroni correction, $**p < 0.01$, $***p < 0.001$. Significance levels for T cells are shown compared to Pediatric Inflamed. Significance levels for all other cell-types are shown compared to Myeloid Predominant. **E)** Ridge plots showing distributions for T-cell subtypes as estimated by

methylation-based deconvolution analysis across immune clusters derived from RNA-seq. In all boxplots, boxes show median and IQR and whiskers represent 1.5 times IQR.

Figure S7. Distribution of clinical parameters across cancer entities. A-B) Barplots showing fraction of gender (A) and race (B) across cancer entities in pedNST. **C)** Boxplot depicting age at diagnosis (in years) across cancer entities in pedNST. Boxes show median and IQR and whiskers represent 1.5 times IQR.

Figure S8. Profiling immune checkpoint genes reveals a subgroup of neuroblastoma with elevated *HAVCR2 and* high *LAG3* expression in Immune Desert. A) Heatmap showing median z-scores of genes specific to B cells, plasma cells and macrophages that were also significantly upregulated in Pediatric Inflamed. **B)** Boxplot showing expression of five targetable immune checkpoint genes in 79 PDX models derived from ATRT, ependymoma, medulloblastoma, neuroblastoma and pedHGG. **C)** Boxplots showing *LAG3* gene expression in the Immune Desert cluster across all cancer types. **D)** Heatmap showing expression of five immune checkpoint genes in two independent datasets, TARGET (top) and Kids First NBL (bottom). **E)** Protein levels of TIM3 and LAG3 in an independent neuroblastoma tissue microarray. Left panel shows staining H-scores obtained from a pathologist's review. Representative images of high TIM3/low LAG3 and low TIM3/low LAG3 are shown on the right. In all boxplots, boxes show median and IQR and whiskers represent 1.5 times IQR.

Figure S9. HLA class I frequencies in pedNST. Barplots showing frequency of HLA-A, B and C across pedNST.

Figure S10. Distribution of predicted peptides across cancer entities and immune clusters. A-B) Boxplots showing predicted strong (A) and weak (B) binding peptides across cancer entities in pedNST**. C-D)** Boxplots depicting predicted weak binding peptides in the pedNST (C) and pedHGG (D) samples across immune clusters**.** Two-sided rank sum test, *p < 0.05. In all boxplots, boxes show median and IQR and whiskers represent 1.5 times IQR.

Figure S11. Validation of T- and B- cell repertoire analysis and distributions across cancer entities. A-B) Scatterplots depicting linear correlation between TCRβ Simpson diversity (A) or richness (B) estimated from RNA-seq and measured by capturing TCR sequences from the same RNA-seq libraries in adult and pediatric cancer samples**. C)** TCRβ estimated Shannon diversity across cancer entities in pedNST ordered by median. **D)** Barplots showing total number of immunoglobulin isotypes normalized by number of samples in each cancer type (top barplot) and isotype fractions (stacked barplot) in each cancer entity. **E)** The gini index for B-cell repertoire across cancer entities in pedNST ordered by median. Circle plots show two craniopharyngioma samples with gini indices of 0.74 (top) and 0.22 (bottom). Each circle is one B-cell clone and circle diameters are proportional to immunoglobulin reads. Blue circles denoted clusters of highly similar immunoglobulin sequences.

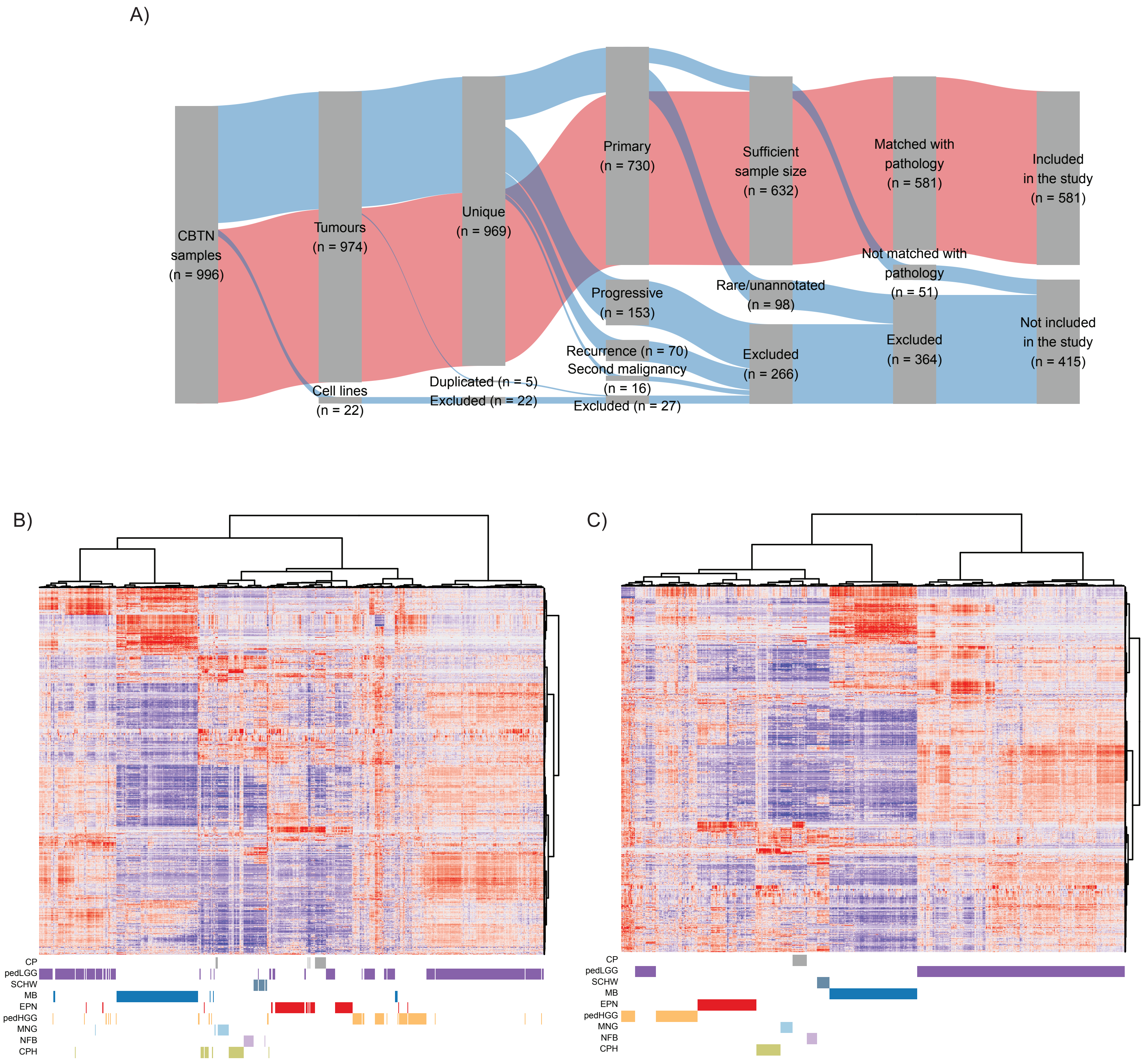
A)
CBTN samples (n = 996)
Tumours (n = 974)
Cell lines (n = 22)
Unique (n = 969)
Duplicated (n = 5)
Excluded (n = 22)
Primary (n = 730)
Progressive (n = 153)
Recurrence (n = 70)
Second malignancy (n = 16)
Excluded (n = 27)
Sufficient sample size (n = 632)
Rare/unannotated (n = 98)
Excluded (n = 266)
Matched with pathology (n = 581)
Not matched with pathology (n = 51)
Excluded (n = 364)
Included in the study (n = 581)
Not included in the study (n = 415)
B)
C)
CP
pedLGG
SCHW
MB
EPN
pedHGG
MNG
NFB
CPH

Figure S1

D)

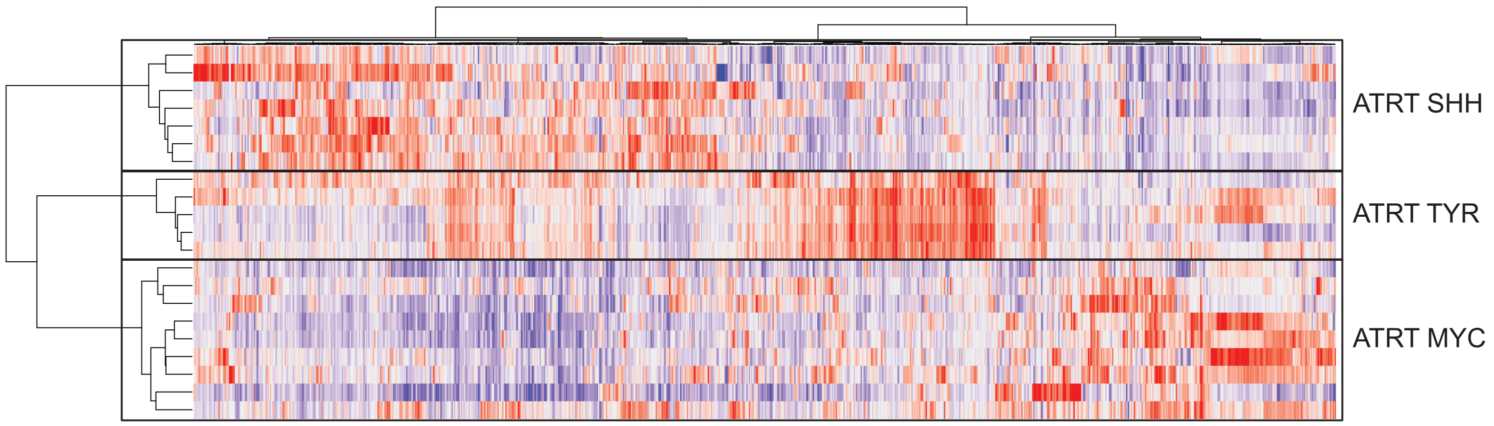

E)

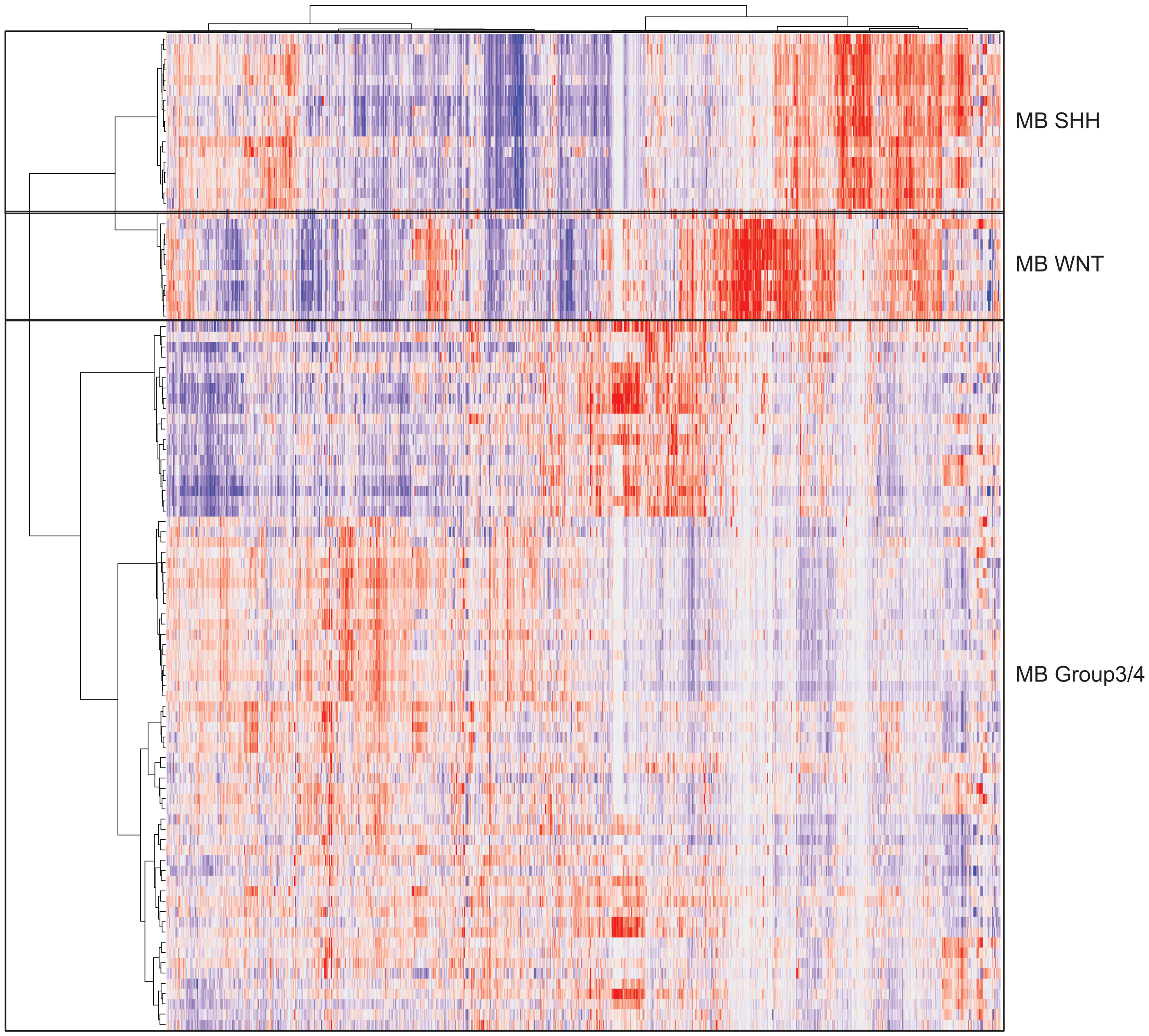

Figure S1

A)

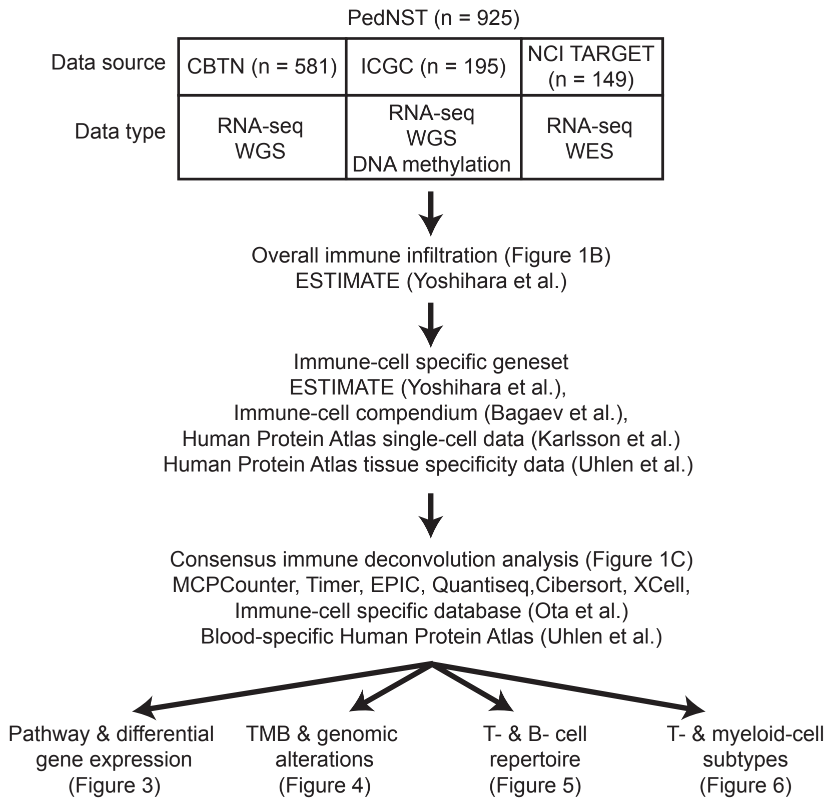
PedNST (n = 925)
Data source
CBTN (n = 581)
ICGC (n = 195)
NCI TARGET (n = 149)
Data type
RNA-seq
WGS
RNA-seq
WGS
DNA methylation
RNA-seq
WES
Overall immune infiltration (Figure 1B)
ESTIMATE (Yoshihara et al.)
Immune-cell specific geneset
ESTIMATE (Yoshihara et al.),
Immune-cell compendium (Bagaev et al.),
Human Protein Atlas single-cell data (Karlsson et al.)
Human Protein Atlas tissue specificity data (Uhlen et al.)
Consensus immune deconvolution analysis (Figure 1C)
MCPCounter, Timer, EPIC, Quantiseq,Cibersort, XCell,
Immune-cell specific database (Ota et al.)
Blood-specific Human Protein Atlas (Uhlen et al.)
Pathway & differential gene expression (Figure 3)
TMB & genomic alterations (Figure 4)
T- & B- cell repertoire (Figure 5)
T- & myeloid-cell subtypes (Figure 6)

B)

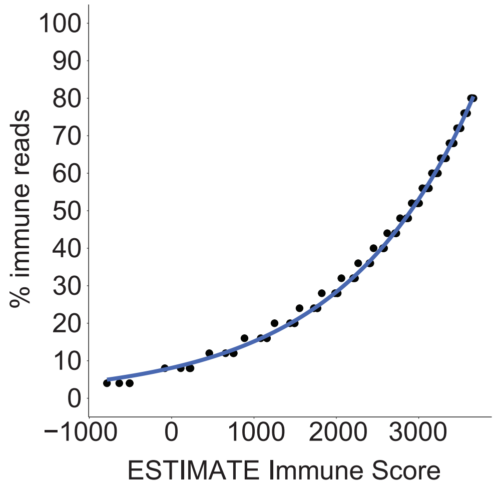
% immune reads
100
90
80
70
60
50
40
30
20
10
0
−1000
0
1000
2000
3000
ESTIMATE Immune Score

Figure S2

A)

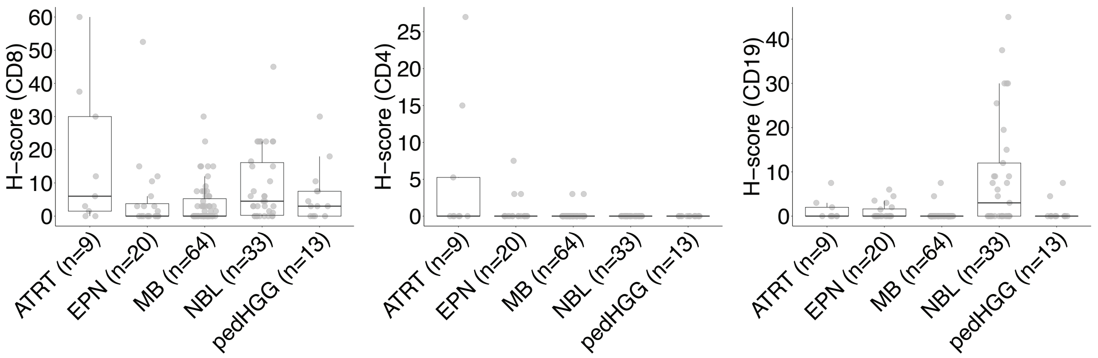
H−score (CD8)
60
50
40
30
20
10
0
ATRT (n=9)
EPN (n=20)
MB (n=64)
NBL (n=33)
pedHGG (n=13)
H−score (CD4)
25
20
15
10
5
0
ATRT (n=9)
EPN (n=20)
MB (n=64)
NBL (n=33)
pedHGG (n=13)
H−score (CD19)
40
30
20
10
0
ATRT (n=9)
EPN (n=20)
MB (n=64)
NBL (n=33)
pedHGG (n=13)

B)

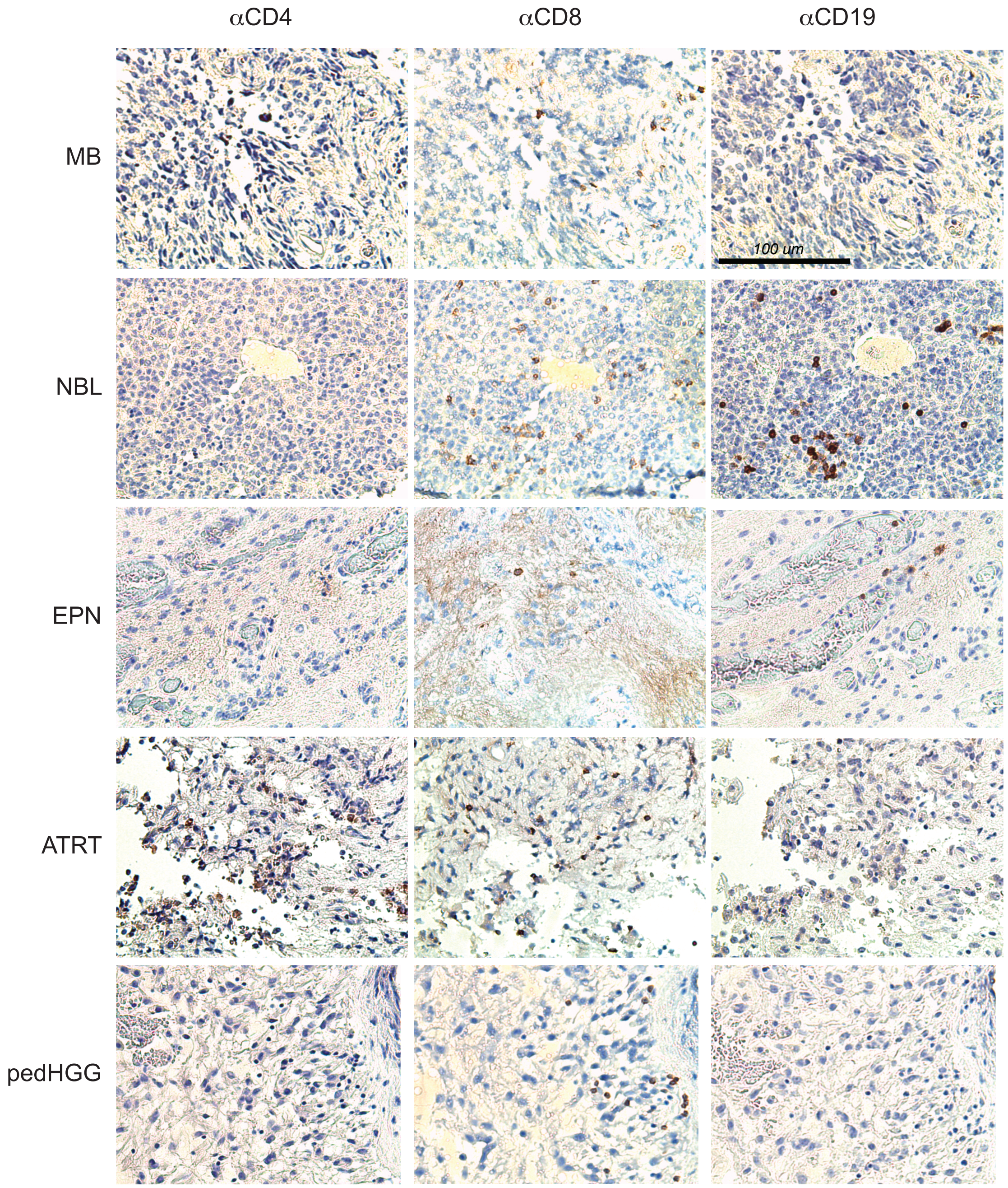
αCD4
αCD8
αCD19
MB
NBL
EPN
ATRT
pedHGG
100 um

Figure S3

A)

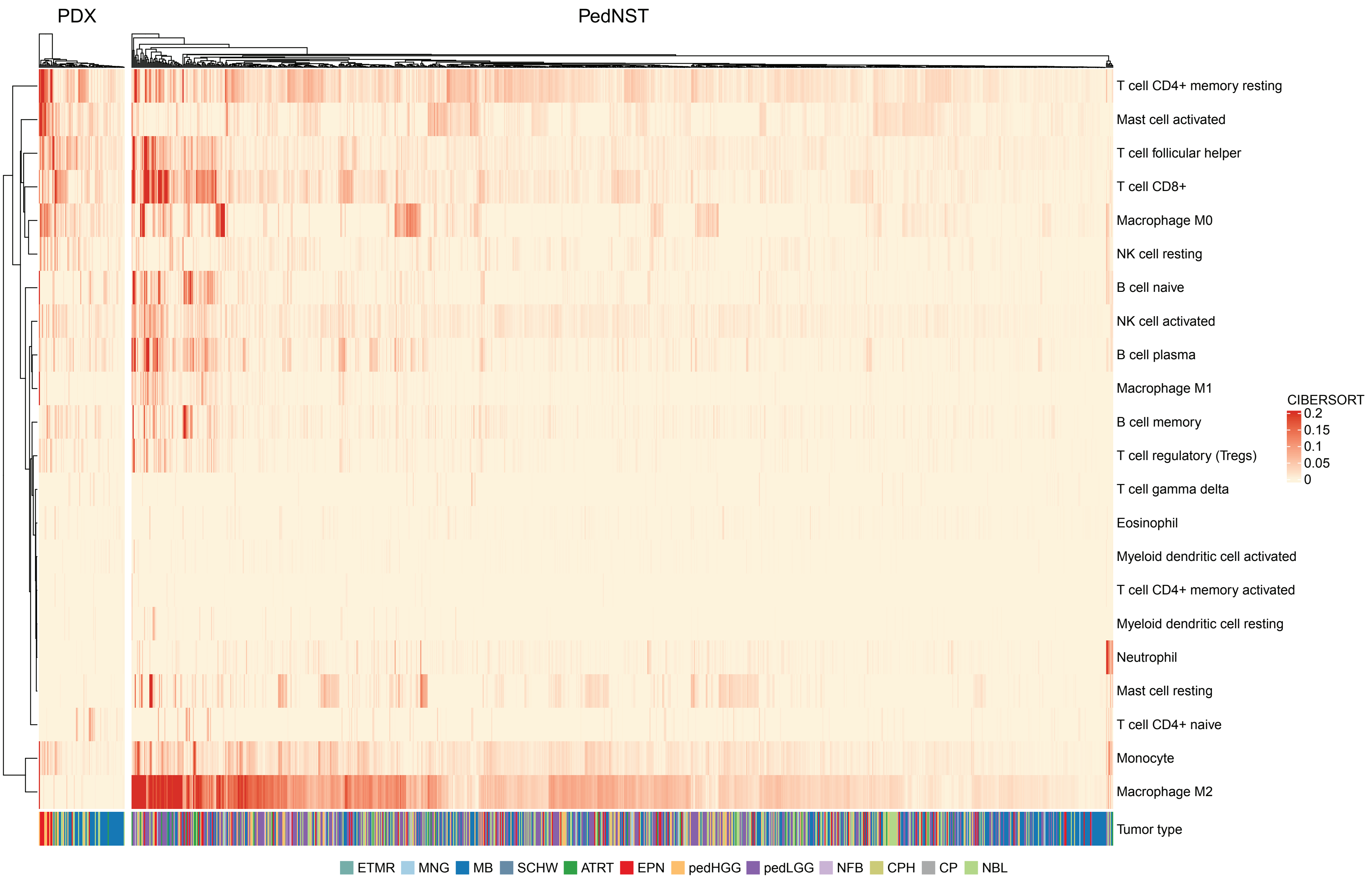
PDX
PedNST
T cell CD4+ memory resting
Mast cell activated
T cell follicular helper
T cell CD8+
Macrophage M0
NK cell resting
B cell naive
NK cell activated
B cell plasma
Macrophage M1
B cell memory
T cell regulatory (Tregs)
T cell gamma delta
Eosinophil
Myeloid dendritic cell activated
T cell CD4+ memory activated
Myeloid dendritic cell resting
Neutrophil
Mast cell resting
T cell CD4+ naive
Monocyte
Macrophage M2
Tumor type
CIBERSORT
0.2
0.15
0.1
0.05
0
ETMR
MNG
MB
SCHW
ATRT
EPN
pedHGG
pedLGG
NFB
CPH
CP
NBL

Figure S4

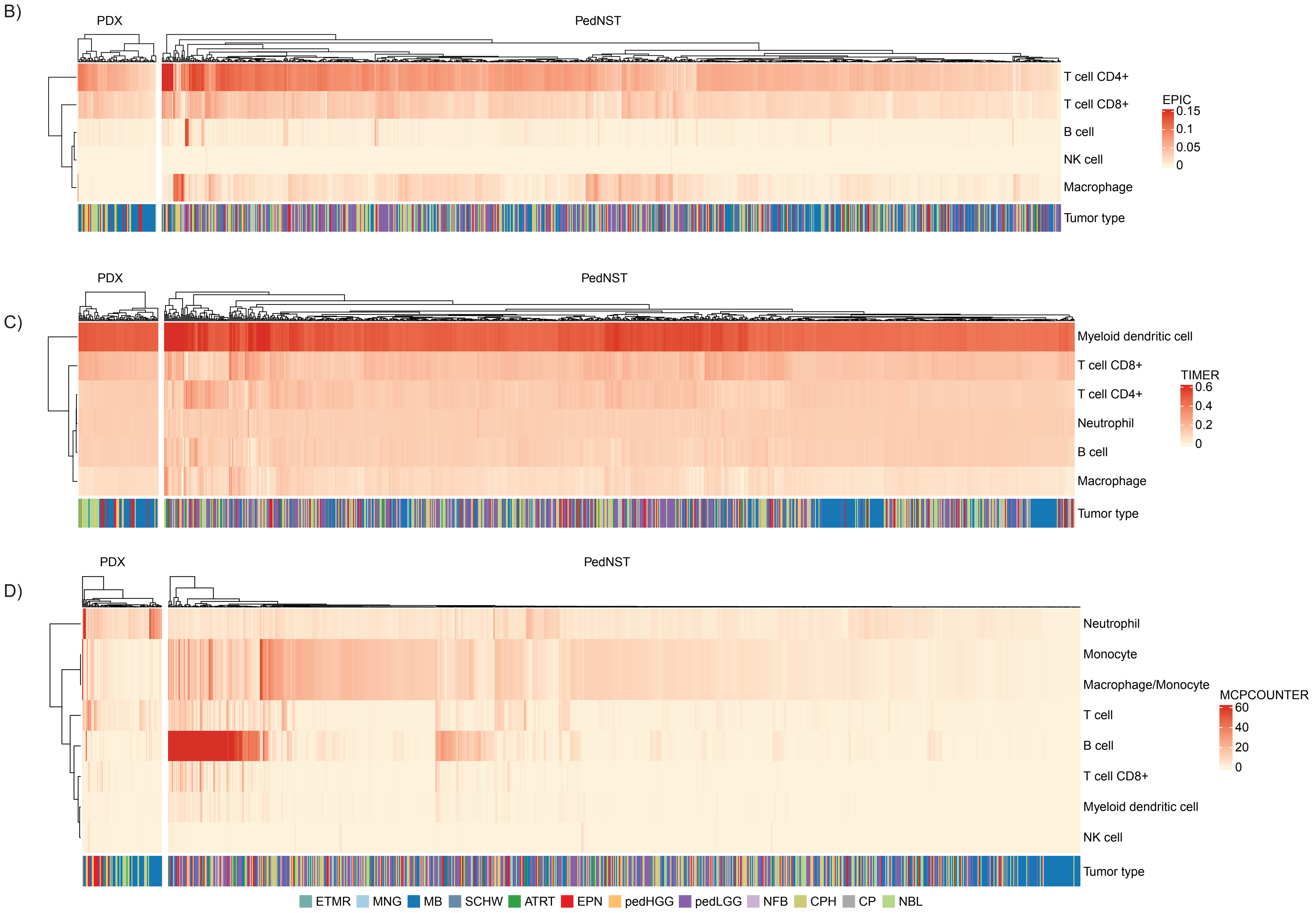
B)
PDX
PedNST
T cell CD4+
T cell CD8+
B cell
NK cell
Macrophage
Tumor type
EPIC
0.15
0.1
0.05
0
C)
PDX
PedNST
Myeloid dendritic cell
T cell CD8+
T cell CD4+
Neutrophil
B cell
Macrophage
Tumor type
TIMER
0.6
0.4
0.2
0
D)
PDX
PedNST
Neutrophil
Monocyte
Macrophage/Monocyte
T cell
B cell
T cell CD8+
Myeloid dendritic cell
NK cell
Tumor type
MCPCOUNTER
60
40
20
0
ETMR MNG MB SCHW ATRT EPN pedHGG pedLGG NFB CPH CP NBL

Figure S4

E)

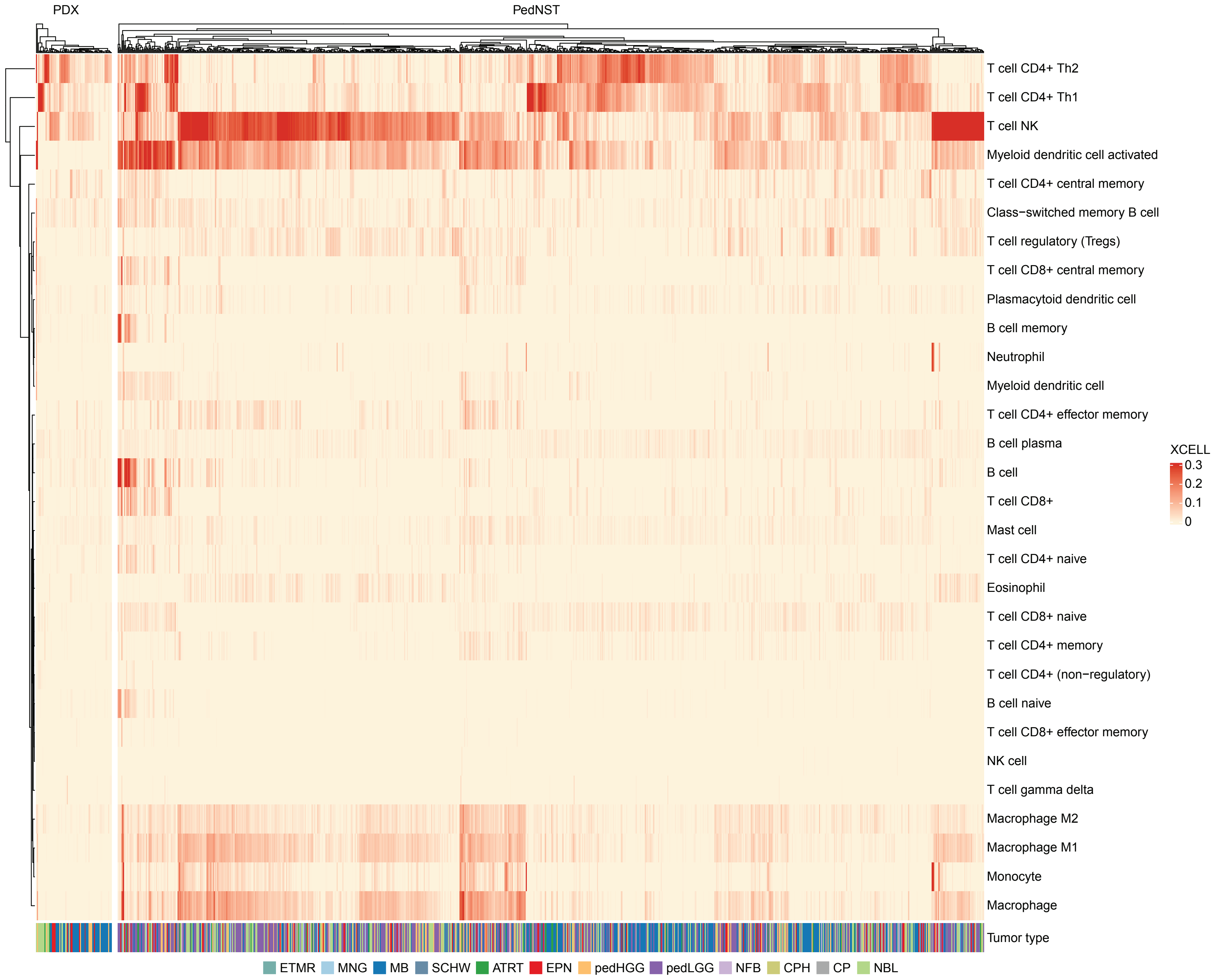
PDX
PedNST
T cell CD4+ Th2
T cell CD4+ Th1
T cell NK
Myeloid dendritic cell activated
T cell CD4+ central memory
Class−switched memory B cell
T cell regulatory (Tregs)
T cell CD8+ central memory
Plasmacytoid dendritic cell
B cell memory
Neutrophil
Myeloid dendritic cell
T cell CD4+ effector memory
B cell plasma
B cell
T cell CD8+
Mast cell
T cell CD4+ naive
Eosinophil
T cell CD8+ naive
T cell CD4+ memory
T cell CD4+ (non−regulatory)
B cell naive
T cell CD8+ effector memory
NK cell
T cell gamma delta
Macrophage M2
Macrophage M1
Monocyte
Macrophage
Tumor type
XCELL
0.3
0.2
0.1
0
ETMR MNG MB SCHW ATRT EPN pedHGG pedLGG NFB CPH CP NBL

Figure S4

F)

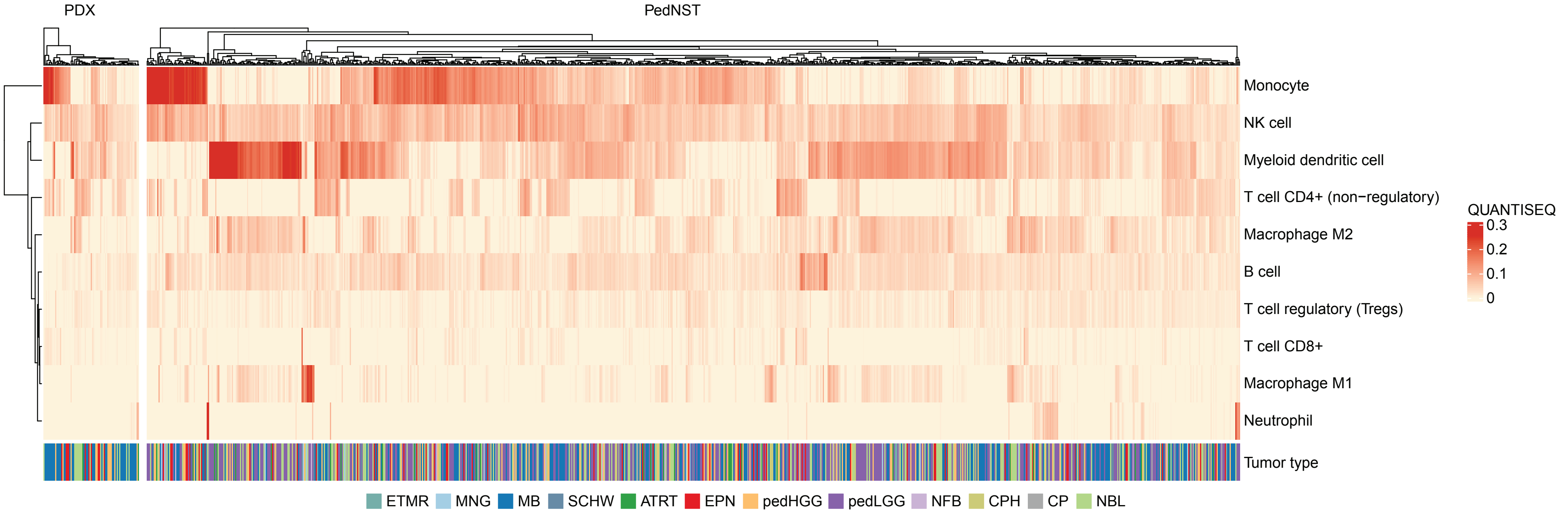
PDX
PedNST
Monocyte
NK cell
Myeloid dendritic cell
T cell CD4+ (non−regulatory)
Macrophage M2
B cell
T cell regulatory (Tregs)
T cell CD8+
Macrophage M1
Neutrophil
Tumor type
QUANTISEQ
0.3
0.2
0.1
0
ETMR
MNG
MB
SCHW
ATRT
EPN
pedHGG
pedLGG
NFB
CPH
CP
NBL

Figure S4

G)

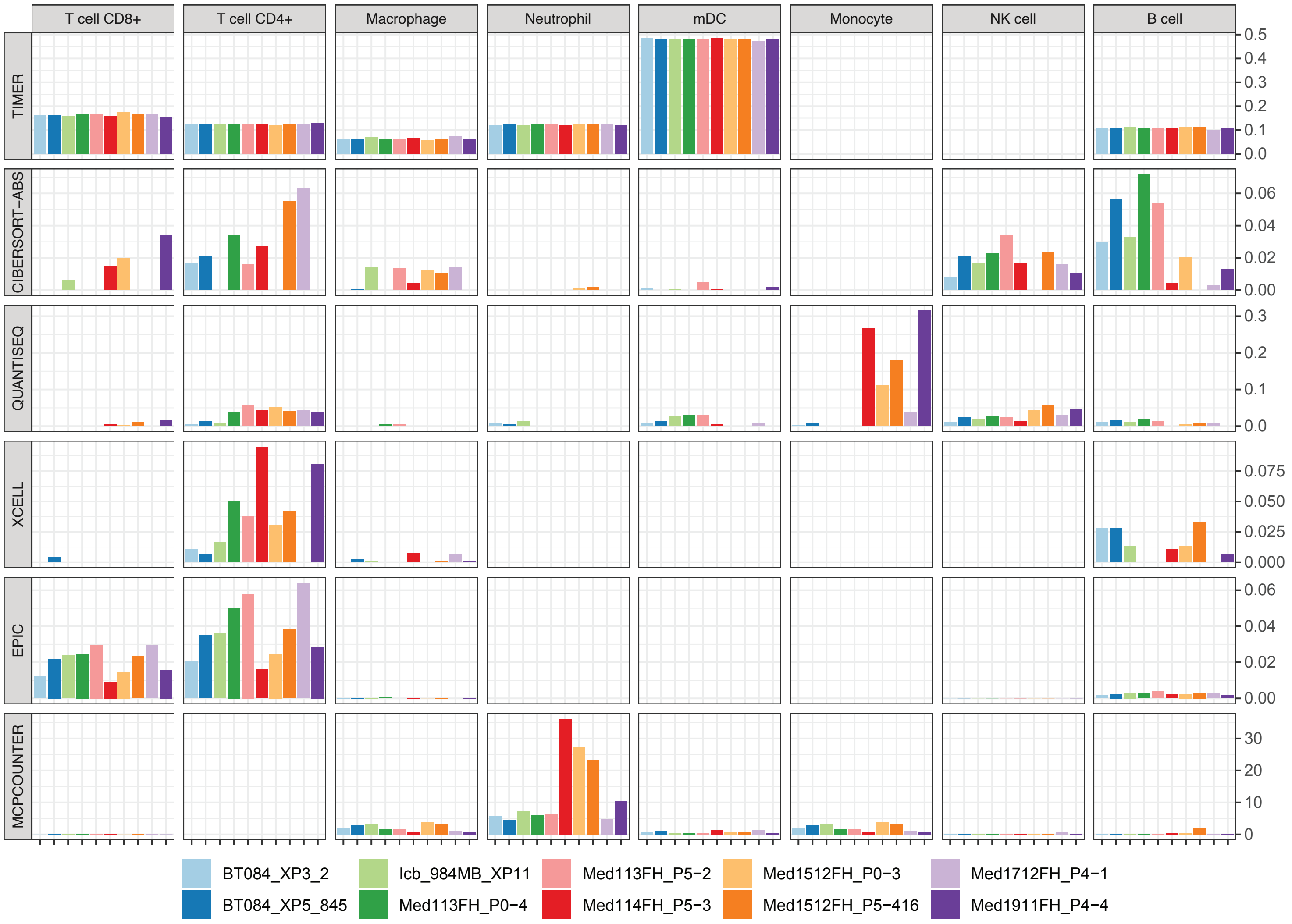

T cell CD8+
T cell CD4+
Macrophage
Neutrophil
mDC
Monocyte
NK cell
B cell
TIMER
CIBERSORT−ABS
QUANTISEQ
XCELL
EPIC
MCPCOUNTER
0.5
0.4
0.3
0.2
0.1
0.0
0.06
0.04
0.02
0.00
0.3
0.2
0.1
0.0
0.075
0.050
0.025
0.000
0.06
0.04
0.02
0.00
30
20
10
0
BT084_XP3_2
BT084_XP5_845
Icb_984MB_XP11
Med113FH_P0−4
Med113FH_P5−2
Med114FH_P5−3
Med1512FH_P0−3
Med1512FH_P5−416
Med1712FH_P4−1
Med1911FH_P4−4

Figure S4

A)

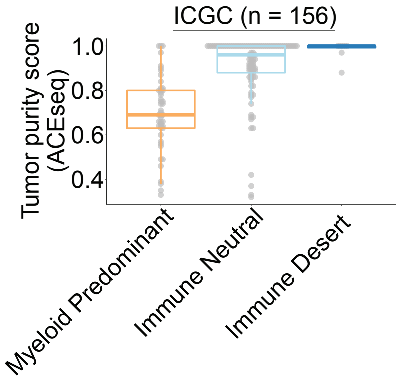

B)

ICGC_GBM6
Tumor cell content = 63%
ESTIMATE ImmuneScore = -71
Myeloid Predominant

ICGC_MB118
Tumor cell content = 94%
ESTIMATE ImmuneScore = -1800
Immune Neutral

ICGC_MB224
Tumor cell content = 100%
ESTIMATE ImmuneScore = -2252
Immune Desert

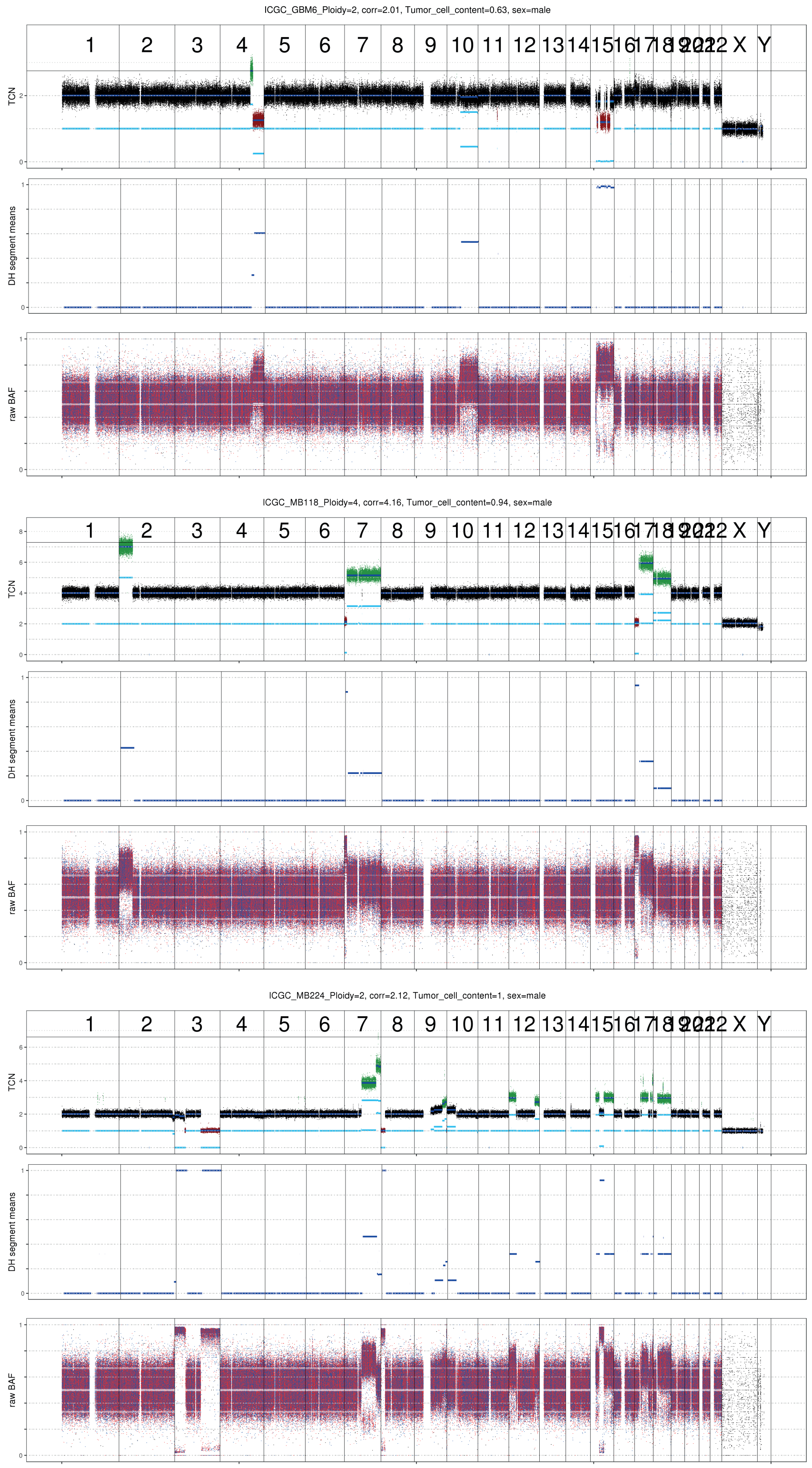

Figure S5

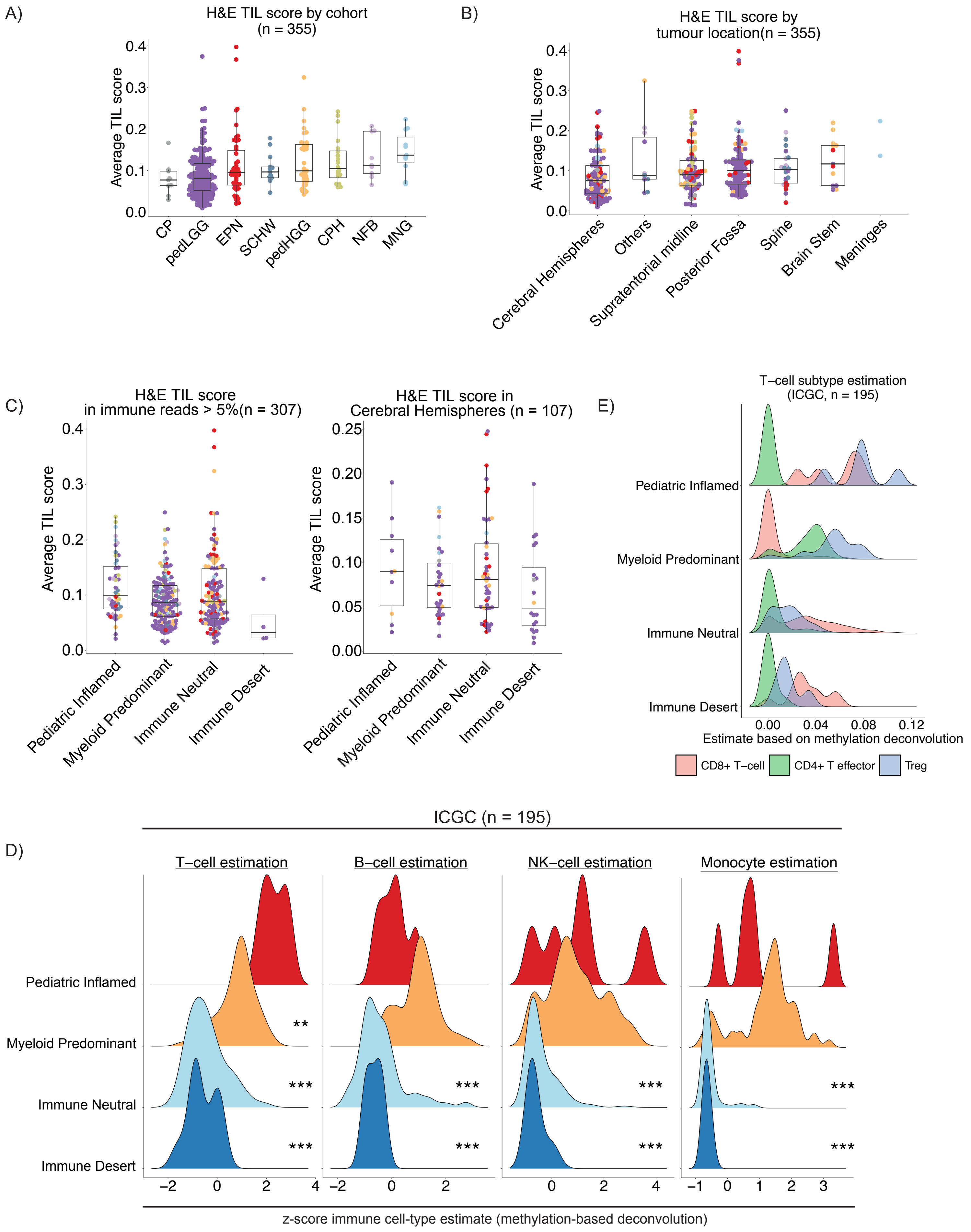
A)
H&E TIL score by cohort
(n = 355)
Average TIL score
0.0
0.1
0.2
0.3
0.4
CP
pedLGG
EPN
SCHW
pedHGG
CPH
NFB
MNG
B)
H&E TIL score by
tumour location(n = 355)
Average TIL score
0.0
0.1
0.2
0.3
0.4
Cerebral Hemispheres
Others
Supratentorial midline
Posterior Fossa
Spine
Brain Stem
Meninges
C)
H&E TIL score
in immune reads > 5%(n = 307)
Average TIL score
0.0
0.1
0.2
0.3
0.4
Pediatric Inflamed
Myeloid Predominant
Immune Neutral
Immune Desert
H&E TIL score in
Cerebral Hemispheres (n = 107)
Average TIL score
0.00
0.05
0.10
0.15
0.20
0.25
Pediatric Inflamed
Myeloid Predominant
Immune Neutral
Immune Desert
E)
T−cell subtype estimation
(ICGC, n = 195)
Pediatric Inflamed
Myeloid Predominant
Immune Neutral
Immune Desert
0.00
0.04
0.08
0.12
Estimate based on methylation deconvolution
CD8+ T−cell
CD4+ T effector
Treg
ICGC (n = 195)
D)
T−cell estimation
B−cell estimation
NK−cell estimation
Monocyte estimation
Pediatric Inflamed
Myeloid Predominant
Immune Neutral
Immune Desert
**
***
***
***
***
***
***
***
***
−2
0
2
4
−2
0
2
0
2
4
−1
0
1
2
3
z-score immune cell-type estimate (methylation-based deconvolution)

Figure S6

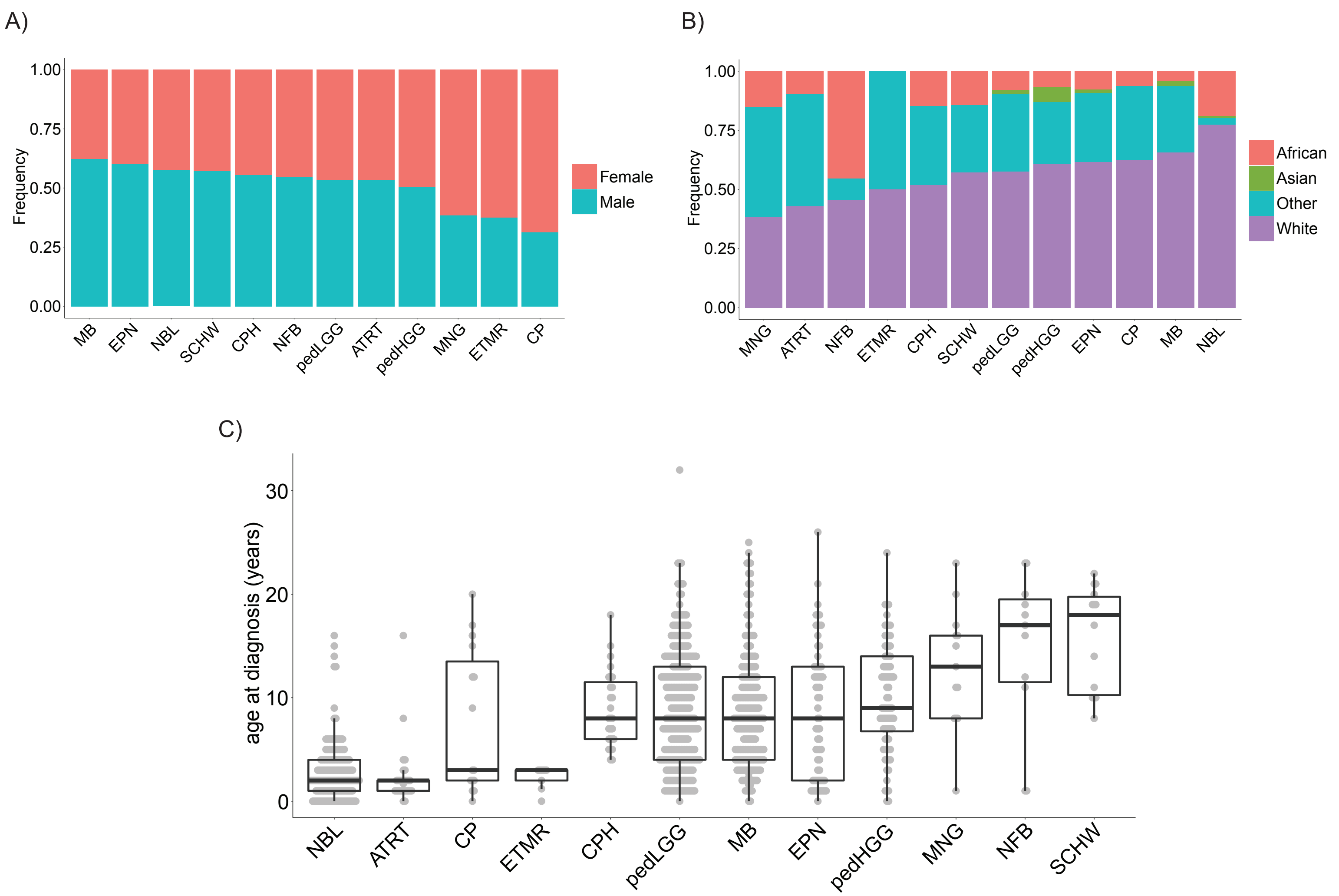
A)
1.00
0.75
0.50
0.25
0.00
Frequency
Female
Male
MB
EPN
NBL
SCHW
CPH
NFB
pedLGG
ATRT
pedHGG
MNG
ETMR
CP
B)
1.00
0.75
0.50
0.25
0.00
Frequency
African
Asian
Other
White
MNG
ATRT
NFB
ETMR
CPH
SCHW
pedLGG
pedHGG
EPN
CP
MB
NBL
C)
30
20
10
0
age at diagnosis (years)
NBL
ATRT
CP
ETMR
CPH
pedLGG
MB
EPN
pedHGG
MNG
NFB
SCHW

Figure S7

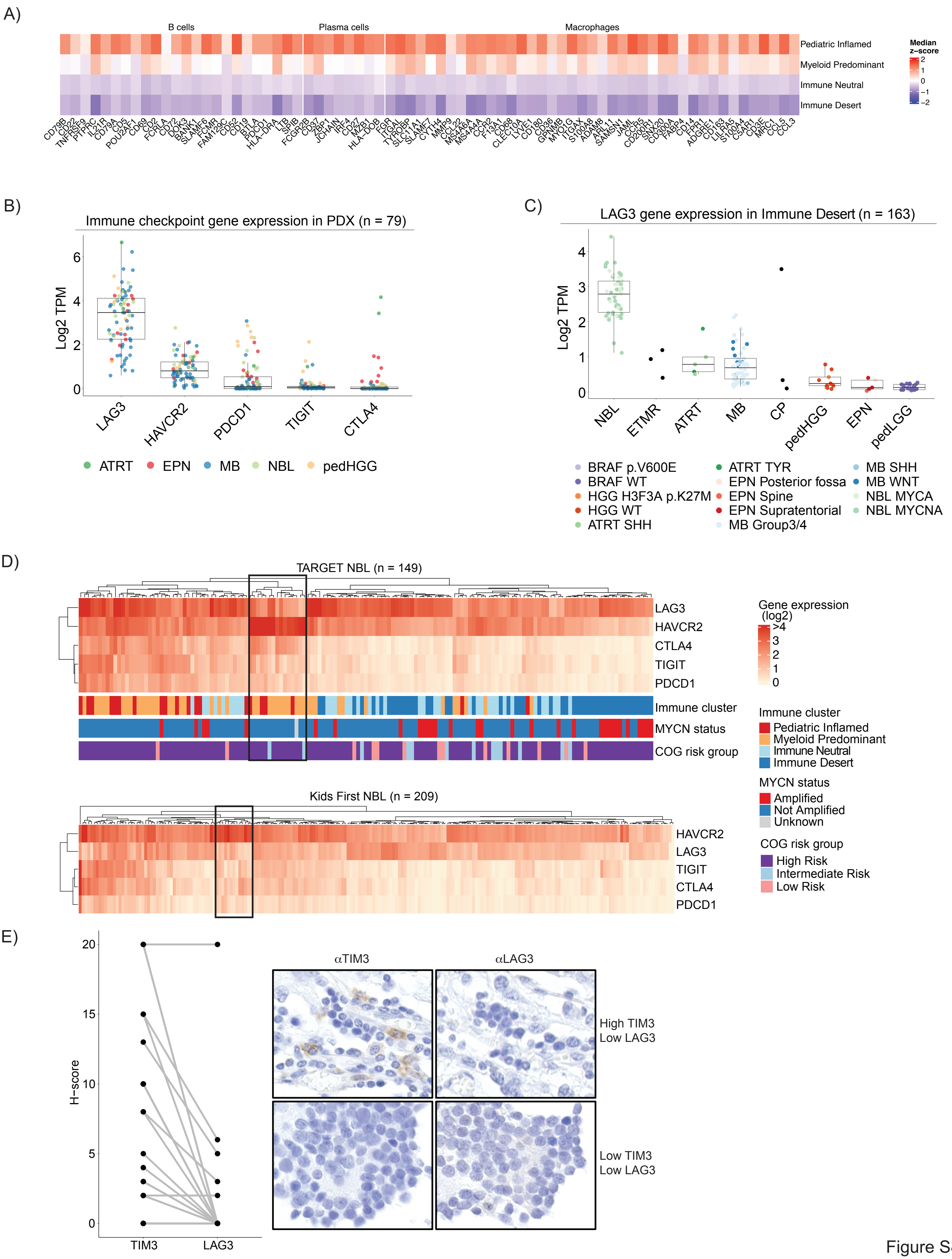
A)
B cells
Plasma cells
Macrophages
Pediatric Inflamed
Myeloid Predominant
Immune Neutral
Immune Desert
Median z-score
2
1
0
−1
−2
B)
Immune checkpoint gene expression in PDX (n = 79)
Log2 TPM
LAG3
HAVCR2
PDCD1
TIGIT
CTLA4
ATRT
EPN
MB
NBL
pedHGG
C)
LAG3 gene expression in Immune Desert (n = 163)
Log2 TPM
NBL
ETMR
ATRT
MB
CP
pedHGG
EPN
pedLGG
BRAF p.V600E
BRAF WT
HGG H3F3A p.K27M
HGG WT
ATRT SHH
ATRT TYR
EPN Posterior fossa
EPN Spine
EPN Supratentorial
MB Group3/4
MB SHH
MB WNT
NBL MYCA
NBL MYCNA
D)
TARGET NBL (n = 149)
LAG3
HAVCR2
CTLA4
TIGIT
PDCD1
Immune cluster
MYCN status
COG risk group
Kids First NBL (n = 209)
HAVCR2
LAG3
TIGIT
CTLA4
PDCD1
Gene expression (log2)
>4
3
2
1
0
Immune cluster
Pediatric Inflamed
Myeloid Predominant
Immune Neutral
Immune Desert
MYCN status
Amplified
Not Amplified
Unknown
COG risk group
High Risk
Intermediate Risk
Low Risk
E)
H-score
TIM3
LAG3
αTIM3
αLAG3
High TIM3
Low LAG3
Low TIM3
Low LAG3

Figure S8

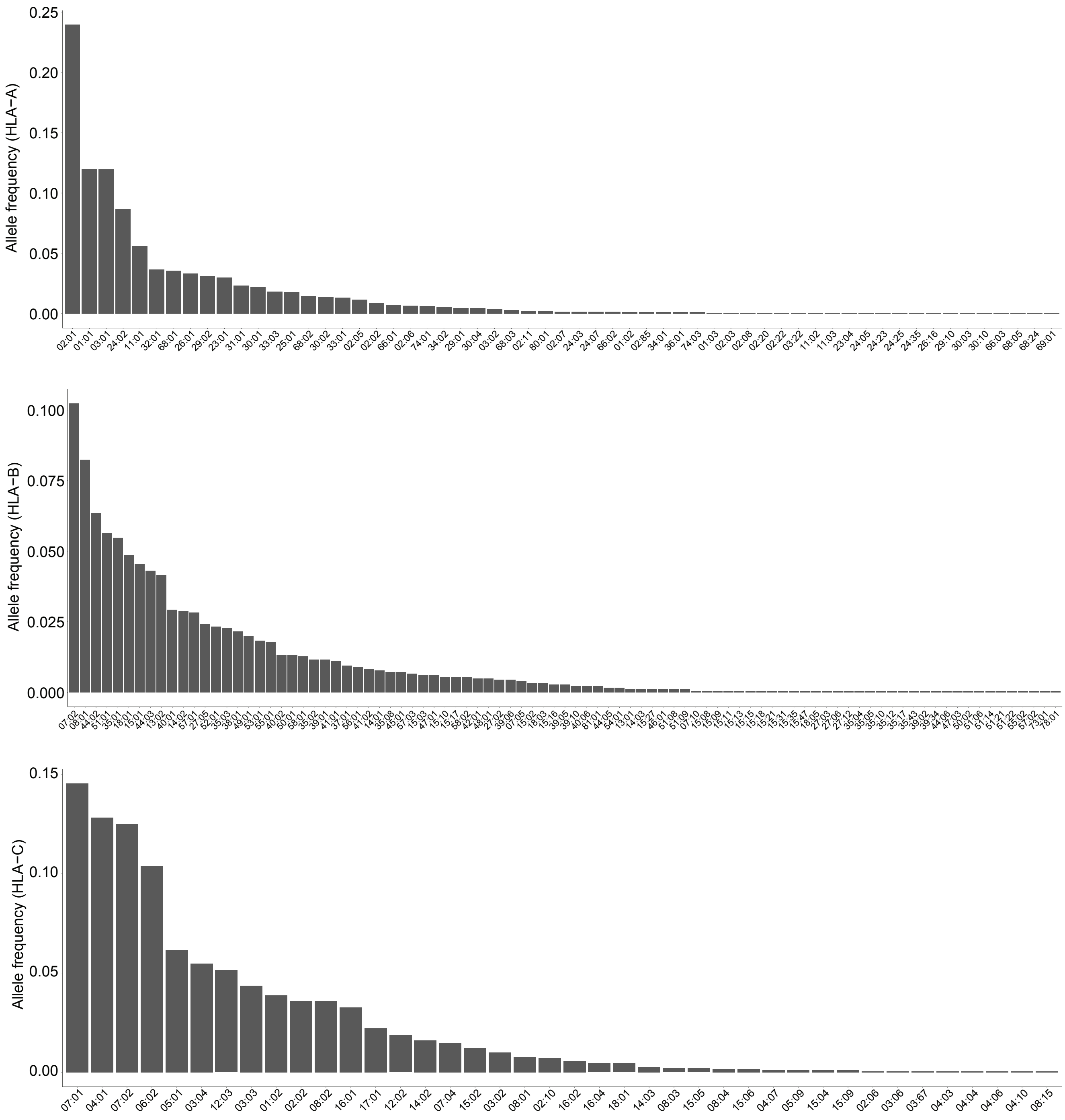
Allele frequency (HLA−A)
0.25
0.20
0.15
0.10
0.05
0.00
02:01 01:01 03:01 24:02 11:01 32:01 68:01 26:01 29:02 23:01 31:01 30:01 33:03 25:01 68:02 30:02 33:01 02:05 02:02 66:01 02:06 74:01 34:02 29:01 30:04 03:02 68:03 02:11 80:01 02:07 24:03 24:07 66:02 01:02 02:85 34:01 36:01 74:03 01:03 02:03 02:08 02:20 02:22 03:22 11:02 11:03 23:04 24:05 24:23 24:25 24:35 26:16 29:10 30:03 30:10 66:03 68:05 68:24 69:01
Allele frequency (HLA−B)
0.100
0.075
0.050
0.025
0.000
Allele frequency (HLA−C)
0.15
0.10
0.05
0.00
07:01 04:01 07:02 06:02 05:01 03:04 12:03 03:03 01:02 02:02 08:02 16:01 17:01 12:02 14:02 07:04 15:02 03:02 08:01 02:10 16:02 16:04 18:01 14:03 08:03 15:05 08:04 15:06 04:07 05:09 15:04 15:09 02:06 03:06 03:67 04:03 04:04 04:06 04:10 08:15

Figure S9

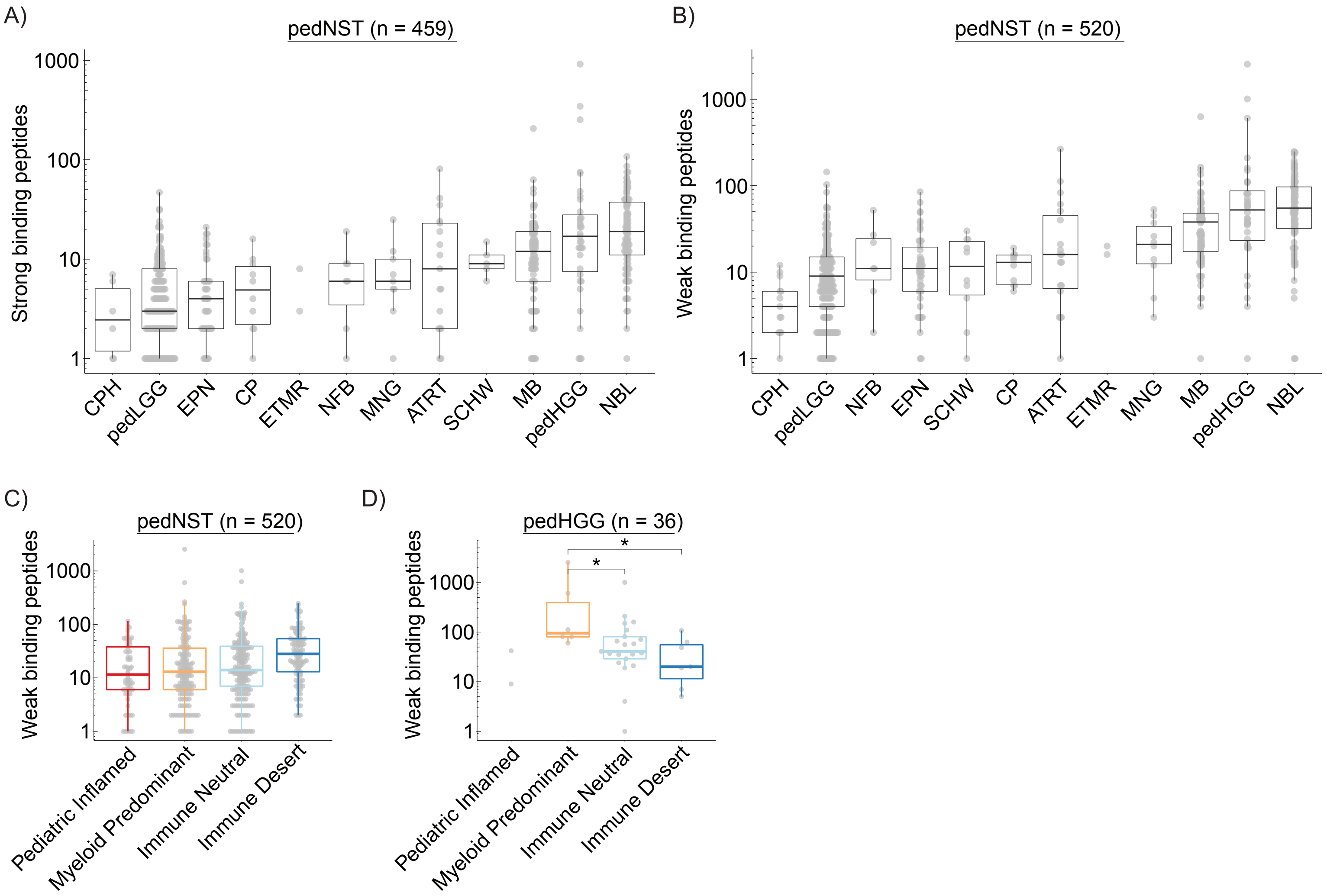
A)
pedNST (n = 459)
Strong binding peptides
1000
100
10
1
CPH
pedLGG
EPN
CP
ETMR
NFB
MNG
ATRT
SCHW
MB
pedHGG
NBL
B)
pedNST (n = 520)
Weak binding peptides
1000
100
10
1
CPH
pedLGG
NFB
EPN
SCHW
CP
ATRT
ETMR
MNG
MB
pedHGG
NBL
C)
pedNST (n = 520)
Weak binding peptides
1000
100
10
1
Pediatric Inflamed
Myeloid Predominant
Immune Neutral
Immune Desert
D)
pedHGG (n = 36)
Weak binding peptides
*
*
1000
100
10
1
Pediatric Inflamed
Myeloid Predominant
Immune Neutral
Immune Desert

Figure S10

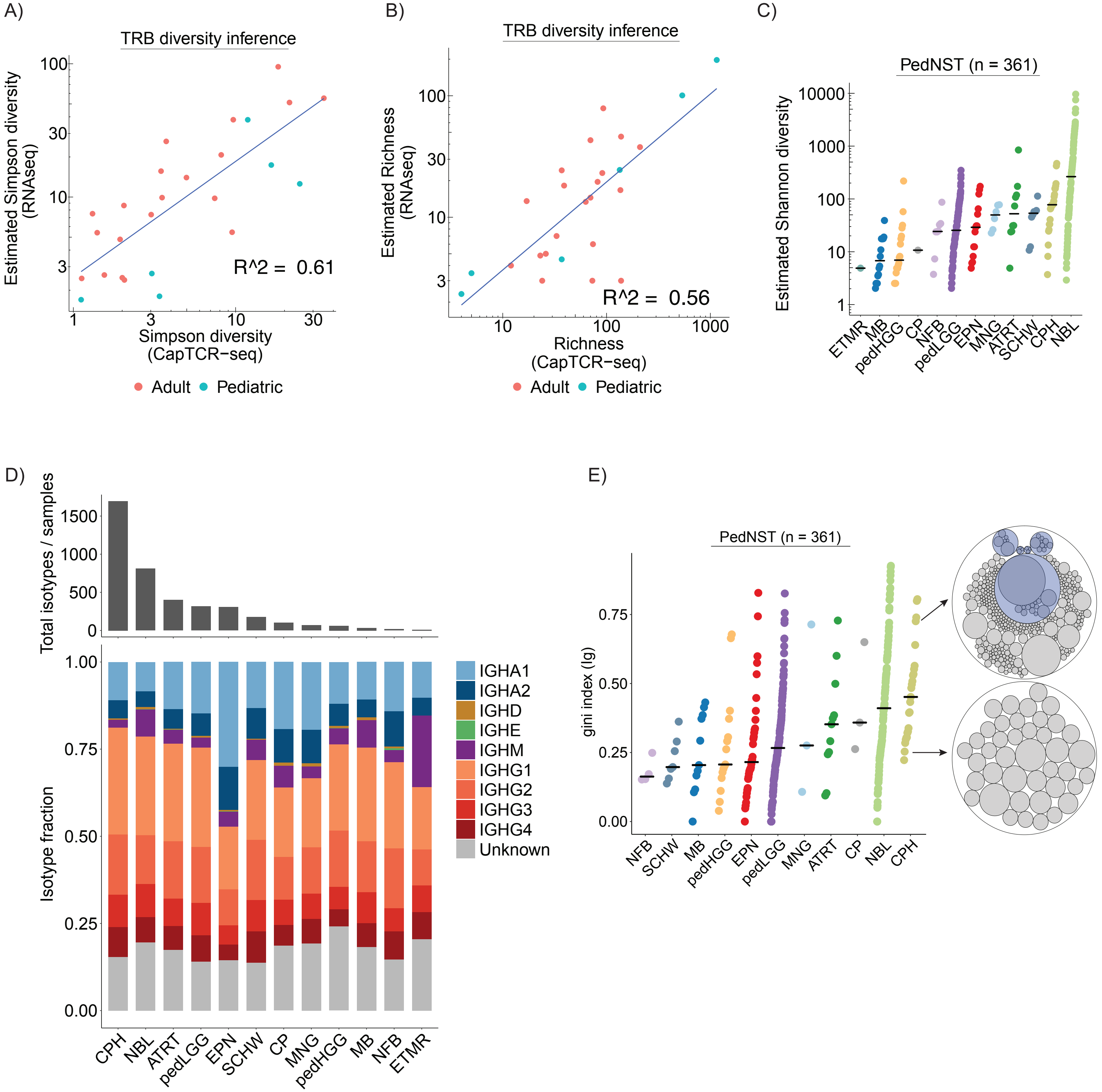
A)
TRB diversity inference
Estimated Simpson diversity (RNAseq)
Simpson diversity (CapTCR−seq)
R^2 = 0.61
Adult
Pediatric
B)
TRB diversity inference
Estimated Richness (RNAseq)
Richness (CapTCR−seq)
R^2 = 0.56
Adult
Pediatric
C)
PedNST (n = 361)
Estimated Shannon diversity
ETMR
MB
pedHGG
CP
NFB
pedLGG
EPN
MNG
ATRT
SCHW
CPH
NBL
D)
Total isotypes / samples
Isotype fraction
IGHA1
IGHA2
IGHD
IGHE
IGHM
IGHG1
IGHG2
IGHG3
IGHG4
Unknown
CPH
NBL
ATRT
pedLGG
EPN
SCHW
CP
MNG
pedHGG
MB
NFB
ETMR
E)
PedNST (n = 361)
gini index (lg)
NFB
SCHW
MB
pedHGG
EPN
pedLGG
MNG
ATRT
CP
NBL
CPH

Figure S11
